# Efficacy and Safety of Landiolol in the Treatment of Tachycardia in Patients With Sepsis and Septic Shock: A Systematic Review and Meta-Analysis

**DOI:** 10.7759/cureus.88004

**Published:** 2025-07-15

**Authors:** Shivraj Paneer Selvam, Norma Nicole Gamarra-Valverde, Andrea Tripoli, Miguel A Samaniego, Juliana Giorgi

**Affiliations:** 1 Medicine, Cardiff University, Cardiff, GBR; 2 Medicine, Universidad Peruana Cayetano Heredia, Lima, PER; 3 Cardiology, San Raffaele Hospital, Milan, ITA; 4 General Medicine, Universidad Autónoma Metropolitana, Mexico City, MEX; 5 Cardiology, Hospital Sirio-Libanes, São Paulo, BRA; 6 Cardiology, Hospital Israelita Albert Einstein, São Paulo, BRA

**Keywords:** beta-blocker, heart rate control, landiolol, sepsis, septic shock, tachyarrhythmia

## Abstract

Landiolol is being investigated for its potential to manage septic shock (SS) and sepsis-related tachyarrhythmias (TA). We performed a systematic review and meta-analysis of three randomized controlled trials (RCTs) involving 473 patients with sepsis or SS, including those with TA, comparing landiolol to standard therapy-controlled (STC) groups. Standard therapy consisted of usual sepsis care ± placebo but excluded β-blockers in the control arms. MEDLINE, Embase, and Cochrane databases were searched for trial data extracted from published reports up to November 2024, excluding non-English reports. Quality assessment was performed per Cochrane recommendations. Risk ratios (RRs) and mean differences (MDs) with 95% confidence intervals (CIs) were pooled across trials to evaluate the outcomes. The primary endpoints included heart rate (HR) at 96 hours, chosen because all three trials consistently recorded HR at this time point, allowing for uniform comparison despite additional time points being reported, and 28-day mortality. Secondary outcomes included atrial fibrillation (AF), hypotension, changes in Sequential Organ Failure Assessment score, and norepinephrine dose. Of the three RCTs, 473 patients were included, with an intervention-to-control arm ratio of approximately 1:1. A lower HR (MD: -6.36; 95% CI: -9.25, -3.47; p < 0.0001; I² = 0%) was observed in the landiolol group compared to the STC. Hypotension (RR: 3.62, 95% CI: 1.37, 9.58; p = 0.010; I² = 5%) was significantly increased in patients who received landiolol when compared to STC, and 28-day mortality showed no significant difference between the groups (RR: 1.07; 95% CI: 0.72, 1.58; p = 0.74; I² = 44%), as did AF (RR: 0.63; 95% CI: 0.25, 1.59; p = 0.33; I² = 8%). Landiolol, a highly selective ultrashort-acting β1-blocker with distinct pharmacokinetic properties from esmolol, reduces HR in patients with sepsis-related TA without significantly affecting 28-day mortality. However, careful monitoring for hypotension is advised, given the absolute risk increase of 8.4% observed in treated patients. However, results should be interpreted cautiously as only three small trials underpin these results. To our knowledge, this is the first MA to focus exclusively on landiolol in this setting, offering drug-specific insights for critical care management.

## Introduction and background

Tachycardia and atrial fibrillation (AF) are common in patients with sepsis and septic shock (SS), often leading to poor prognoses [1]. Managing these arrhythmias is challenging due to the ineffectiveness or contraindication of conventional treatments. Landiolol, an ultra-short-acting β1-selective β-blocker, is being investigated for its potential to manage sepsis or SS-related tachyarrhythmias (TA).

SS, a severe manifestation of sepsis, frequently leads to systemic inflammation, hemodynamic instability, and an elevated heart rate (HR) [2]. Persistent tachycardia (HR ≥ 95 beats per minute (bpm)) [3-5] in patients with SS has been linked to poor outcomes [1], with mortality rates being greater than 70% in some reported studies [4]. The 95-bpm threshold was chosen based on prior evidence that HR reduction below this level is associated with improved survival, with a range of 80-94 bpm considered optimal for balancing cardiac function and systemic hemodynamics [3-5]. Hence, β-blockers such as landiolol, an ultra-short-acting, highly β1-selective antagonist, have been explored as a therapeutic approach to lower HR and potentially enhance outcomes. This meta-analysis (MA) compiles data from three randomized controlled trials (RCTs) [3-5] to assess the efficacy and safety of landiolol in SS and sepsis-related TA.

Sepsis, a life-threatening condition, results from a dysregulated host response to infection, causing organ dysfunction and systemic inflammation. When sepsis progresses to SS, it is marked by severe circulatory, cellular, and metabolic abnormalities, including persistent hypotension that requires vasopressor therapy and serum lactate levels exceeding 18 mg/dL despite adequate fluid resuscitation [6]. SS impacts nearly one-third of patients with sepsis and has a high mortality rate, often exceeding 40%, due to its complex interplay of immune, hemodynamic, and metabolic dysfunctions [2].

In SS, the excessive release of catecholamines, such as norepinephrine and epinephrine, can trigger sympathetic overstimulation, tachycardia, and cardiovascular strain, which further impair preload, coronary perfusion, and organ function [7]. Significantly, persistent tachycardia can impair diastolic filling and reduce stroke volume, ultimately compromising cardiac output, especially in patients with already impaired ventricular function [8]. Persistent tachycardia is a poor prognostic factor, as it exacerbates myocardial oxygen consumption and reduces cardiac efficiency [8]. Addressing this pathophysiological cascade has led to the exploration of β1-adrenergic receptor blockers, such as landiolol, as adjunctive therapies.

Landiolol is an ultra-short-acting, highly selective β1-blocker that exerts its effects by competitively antagonizing β1-adrenergic receptors in the myocardium, thereby reducing HR without significantly compromising cardiac output or blood pressure. Compared to esmolol, another β1-selective blocker, landiolol demonstrates superior β1-receptor selectivity, lower adverse inotropic effects, and a shorter half-life of approximately four minutes, making it an ideal candidate for precise titration in critically ill patients [9]. Beyond HR control, landiolol may also attenuate the detrimental effects of sympathetic overdrive, including inflammatory responses, microcirculatory dysfunction, and myocardial oxygen imbalance, with potential implications for improving sepsis-related outcomes [10]. These unique pharmacological features make landiolol relevant for HR management in SS, justifying this MA.

International guidelines currently recommend intravenous fluids as first-line therapy for sepsis. They are associated with vasopressors in SS but provide limited evidence on HR management despite evidence linking persistent tachycardia with worse outcomes [11]. The excessive release of catecholamines during sepsis contributes to sympathetic overactivity, thus impairing cardiac performance and increasing mortality [3,5]. Conventional antiarrhythmics such as amiodarone or non-selective β-blockers are often avoided due to risks such as hypotension, negative inotropy, and QT prolongation [5]. In contrast, selective β1 blockers like landiolol may offer better HR control with minimal impact on blood pressure or contractility and may improve cardiac output, lactate clearance, and organ function [3,5].

On the other hand, in comparison with esmolol, landiolol has an ultra-short half-life (four minutes versus esmolol’s nine minutes) and higher β1-selectivity [3]. These pharmacologic properties reduce the likelihood of adverse inotropic effects and hypotension, thus making landiolol a better option for use in hemodynamically unstable patients with sepsis [3,5]. Unlike other β-blockers that are less selective or specific, landiolol allows for precise titration and rapid reversal if required, enabling better hemodynamic control without compromising perfusion [3,5,11].

**Figure 1:**
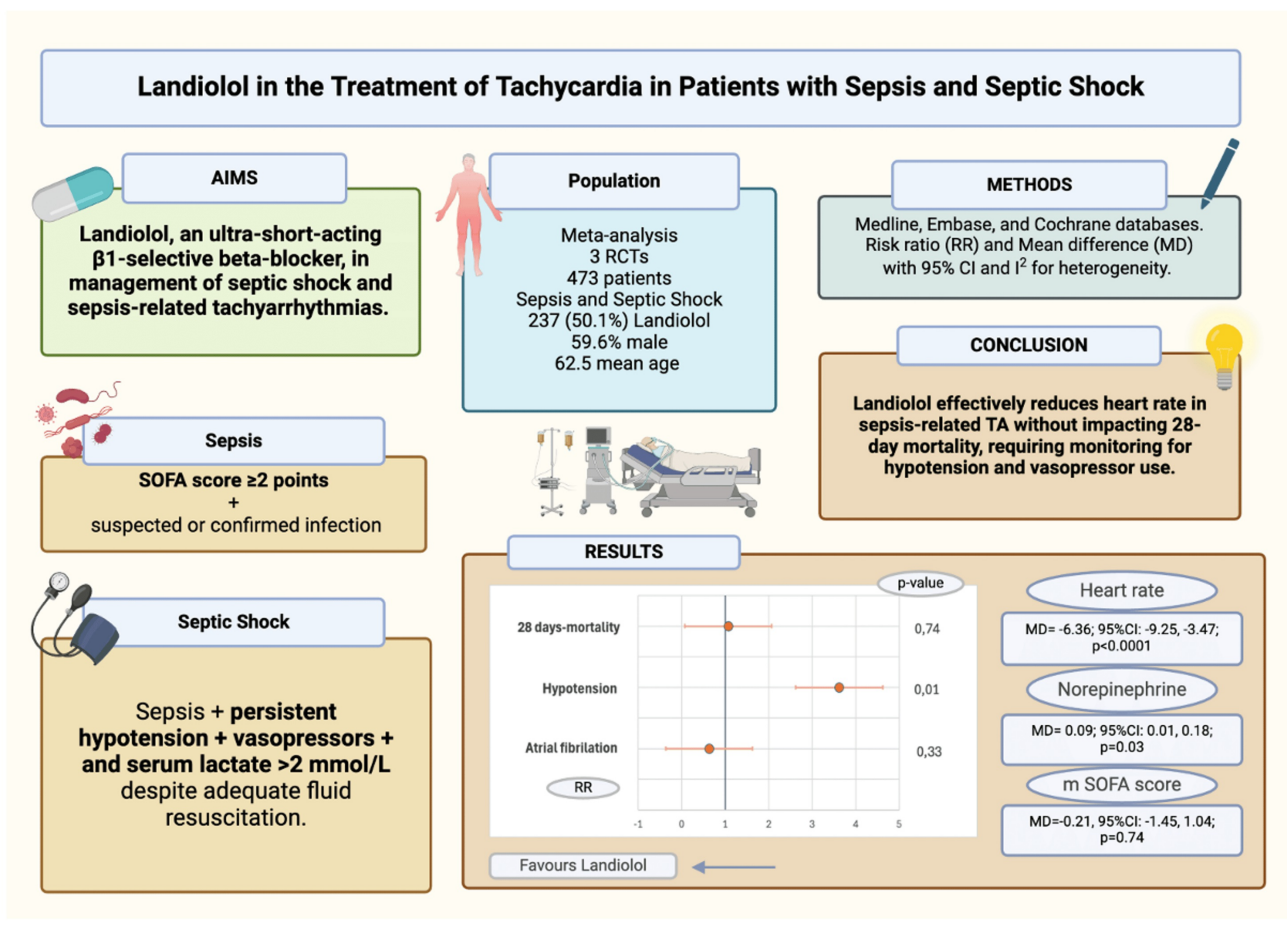
Graphical abstract TA: tachyarrhythmia, RCT: randomized controlled trial, SOFA: sequential organ failure assessment, RR: risk ratio, MD: mean difference, CI: confidence interval, m SOFA: modified sequential organ failure assessment Image Credit: Authors. Made with BioRender. Data were obtained from studies [3-5].

This introduction sets the stage for investigating the efficacy and safety of landiolol (intervention) in reducing 28-day mortality and adverse events like hypotension (outcomes) compared to standard of care (comparator) in patients with sepsis or SS and persistent tachycardia (population). While previous systematic reviews (SR) and MAs have evaluated β-blockers more broadly in sepsis, including agents like esmolol, which has proven to lower HR, improve stroke volume, and reduce vasopressor requirements [8], they have not explored differences in pharmacological profiles across agents. To our knowledge, this is the first MA to focus exclusively on landiolol. Hence, by isolating landiolol, which differs from other agents in its ultra-short half-life and high β1-selectivity, we aim to provide drug-specific insights into its therapeutic role and safety profile in critically ill patients with sepsis and SS. A graphical overview of the study's rationale, methodology, and key findings is illustrated in Figure 1.

## Review

Methods

This SR and MA were performed and reported by the Cochrane Collaboration Handbook for Systematic Review of Interventions and the Preferred Reporting Items for Systematic Reviews and Meta-Analysis (PRISMA) statement guidelines [12]. The protocol for this research was submitted to the International Prospective Register of Systematic Reviews (PROSPERO) with registration number CRD42024615659.

Search Strategy

A systematic search was conducted across multiple databases, including MEDLINE, Cochrane, and Embase, to identify studies published from inception up to November 2024. Non-English papers and grey literature were excluded. The following search terms were utilized: "landiolol", "high heart rate", "sepsis", "septic-shock", "tachyarrhythmia", "atrial fibrillation", and "arrhythmia". The full search strategy, including all search terms and Boolean operators, is provided in the Appendices.

Eligibility Criteria

Inclusion in this MA was restricted to studies that met the following eligibility criteria: (1) RCTs, (2) comparing landiolol to standard therapy-controlled (STC) groups; standard therapy consisted of usual sepsis care ± placebo but excluded β-blockers in the control arms; (3) patients documented with high cardiac rate (HR ≥ 95 bpm). Exclusion criteria are the following: (1) studies that lack a control group, (2) studies that have overlapping patient populations, and (3) studies that used β-blockers other than landiolol as the intervention and that have head-to-head comparisons of β-blockers. In addition, studies were included only if they reported at least one outcome of interest. The references from all included studies, previous SRs, and MAs were also searched manually for any additional studies. All observational studies, animal studies, reviews, and trials without relevant clinical outcomes were excluded.

Data Extraction

Two authors (S.S. and N.M.) independently extracted the data from databases. Duplicates were removed with the Rayyan software (Qatar Computing Research Institute (QCRI), Doha, Qatar). All studies meeting the predefined inclusion criteria were retained for review. Any uncertainties or discrepancies were resolved through consensus-based discussion. An additional reviewer was available if needed.

Risk of Bias Assessment

We evaluated the risk of bias in randomized studies using the Cochrane risk of bias assessment tool version 2 (RoB 2, Cochrane Collaboration, London, UK). Two independent authors completed the risk of bias assessment (S.S., N.M.). Disagreements were resolved through a consensus after discussing the reasons for the discrepancy. Publication bias was investigated using a funnel-plot analysis of point estimates and study weights. However, with only three trials, funnel plots are statistically uninformative. Leave-one-out sensitivity analyses were performed to ensure that the results were not dependent on a single study.

Statistical Analysis

The treatment effects for binary outcomes were assessed using risk ratios (RR), calculated with the Mantel-Haenszel method, and 95% confidence intervals (CIs) were reported. Mean differences (MDs) were used to compare results for continuous outcomes. Heterogeneity across studies was assessed using the Cochrane Q-test and I² statistics, with a p-value < 0.10 and I² > 50% indicating substantial heterogeneity. For endpoints with high heterogeneity, a random-effects model was applied based on recommendations from the Cochrane Collaboration handbook for systematic reviews of interventions. All statistical analyses were performed using Review Manager (RevMan) version 5.4 (Cochrane Collaboration, London, UK) [13].

Results

Study Selection

**Figure 2:**
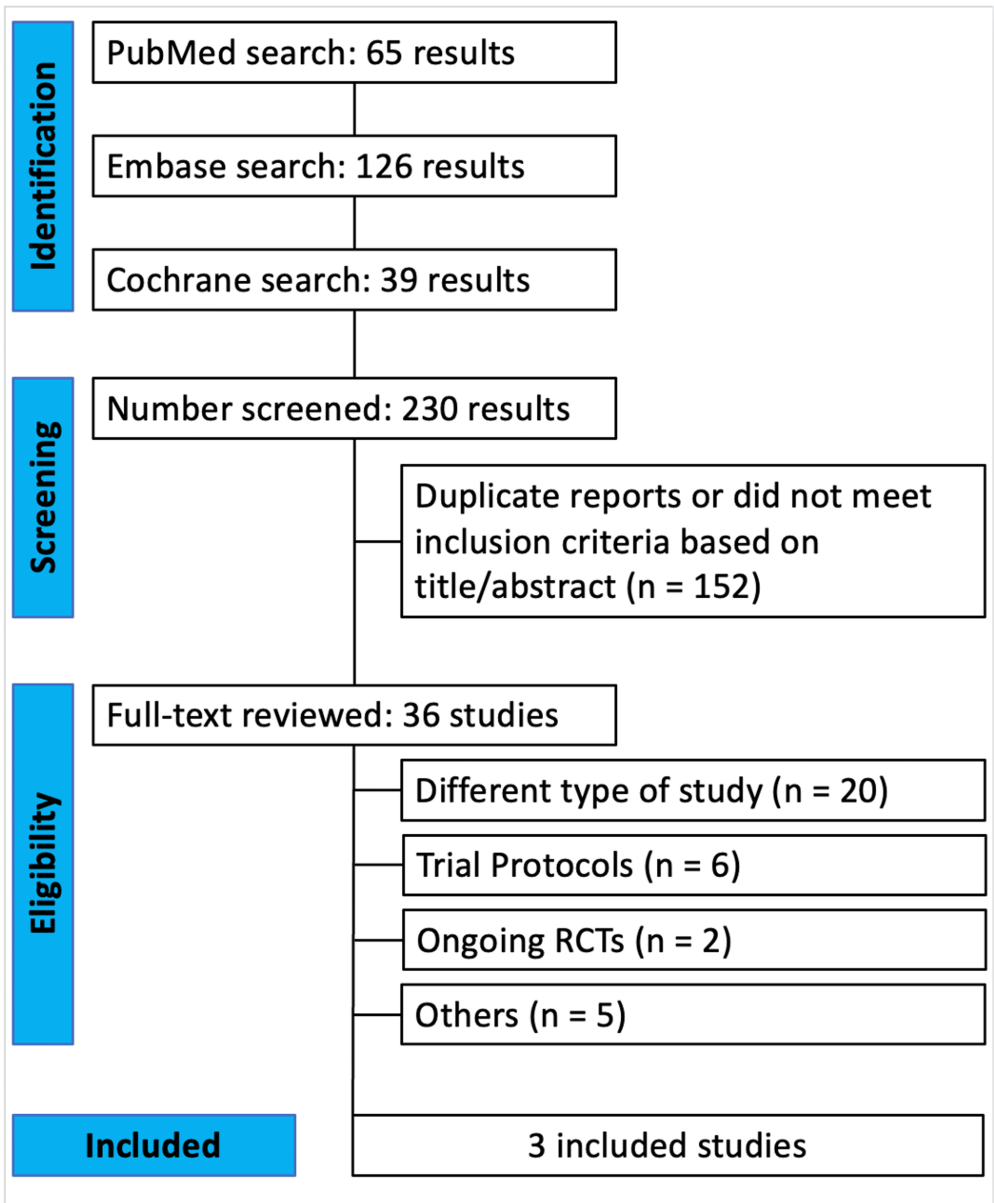
PRISMA flow diagram of study screening and selection PRISMA: Preferred Reporting Items for Systematic Reviews and Meta-Analysis, RCTs: randomized controlled trials Developed based on PRISMA guidelines in [12].

An initial search identified 230 studies (Figure 2). After removing duplicates and excluding ineligible studies, 36 studies were selected for full-text screening. Full-text screening was done manually by the two authors stated above. Three studies met the eligibility criteria and were included in the final review. All studies included were RCTs, involving a total of 473 patients.

Study Characteristics

Of the 473 patients, 237 patients (50.1%) received landiolol. The mean age of patients was 62.5 years, with 282 (59.6%) being men. All studies included adult intensive care unit (ICU) patients with sepsis or SS and persistent tachycardia. The mean HR at baseline ranged from 110.6 bpm to 117.5 bpm, and Sequential Organ Failure Assessment (SOFA) scores averaged between 10.0 and 12.6. Detailed study characteristics are presented in Table 1.

**Table 1:** Baseline characteristics of included studies Data are presented as means unless stated otherwise. L: landiolol, ST: standard treatment, SS: septic shock, AF: atrial fibrillation, HR: heart rate, MAP: mean arterial Pressure, SOFA: Sequential Organ Failure Assessment, a: beats per minute, b: μg/kg/min, c: mm Hg, d: mmol/L, y: years

Study	Patients ^n^, L/ST	Male ^n^, L/ST	Age^y^, L/ST	SS ^n^, L/ST	AF ^n^, L/ST	SOFA score, L/ST	HR ^a^, L/ST	Norepinephrine ^b^, L/ST	MAP ^c^, L/ST	Lactate ^d^, L/ST
J-LAND 3S 2020 [5]	76/75	52/38	67.8/66.4	69/68	17/12	10.0/10.1	117.4/117.6	0.2/0.2	84.1/81.8	3.3/3.2
STRESS-L 2023 [4]	63/63	37/37	55.9/55.3	63/63	7/8	10.1/10.3	110.6/114.1	0.37/0.36	73.0/72.3	4.6/4.5
LANDI-SEP 2024 [3]	98/98	63/55	64.4/65.2	98/98	26/24	12.6/12.1	116.0/114.2	0.51/0.52	78.6/79	4.0/3.9

Endpoints

Outcomes included 28-day all-cause mortality, hypotension, HR at 96 hours, incidence of AF, modified SOFA score, and norepinephrine doses.

Risk of Bias Within Studies

Following the risk of bias assessment, we identified that the STRESS-L trial [4] was associated with a high risk of bias due to deviations from the intended interventions, the selection of reported results, and overall risk (Table 2).

**Table 2:** Risk of bias summary for randomized studies using RoB 2

Study	Bias from the randomization process	Bias due to deviations from intended interventions	Bias due to missing outcome data	Bias in the measurement of the outcomes	Bias in the selection of the reported result	Overall risk of bias
STRESS-L, 2023 [4]	Low	High	Low	Low	High	High
Landi-SEP, 2024 [3]	Low	Some concerns	Low	Some concerns	Low	Some concerns
J-Land 3S, 2020 [5]	Low	Some concerns	Low	Low	Low	Some concerns

Primary Outcomes

**Figure 3:**
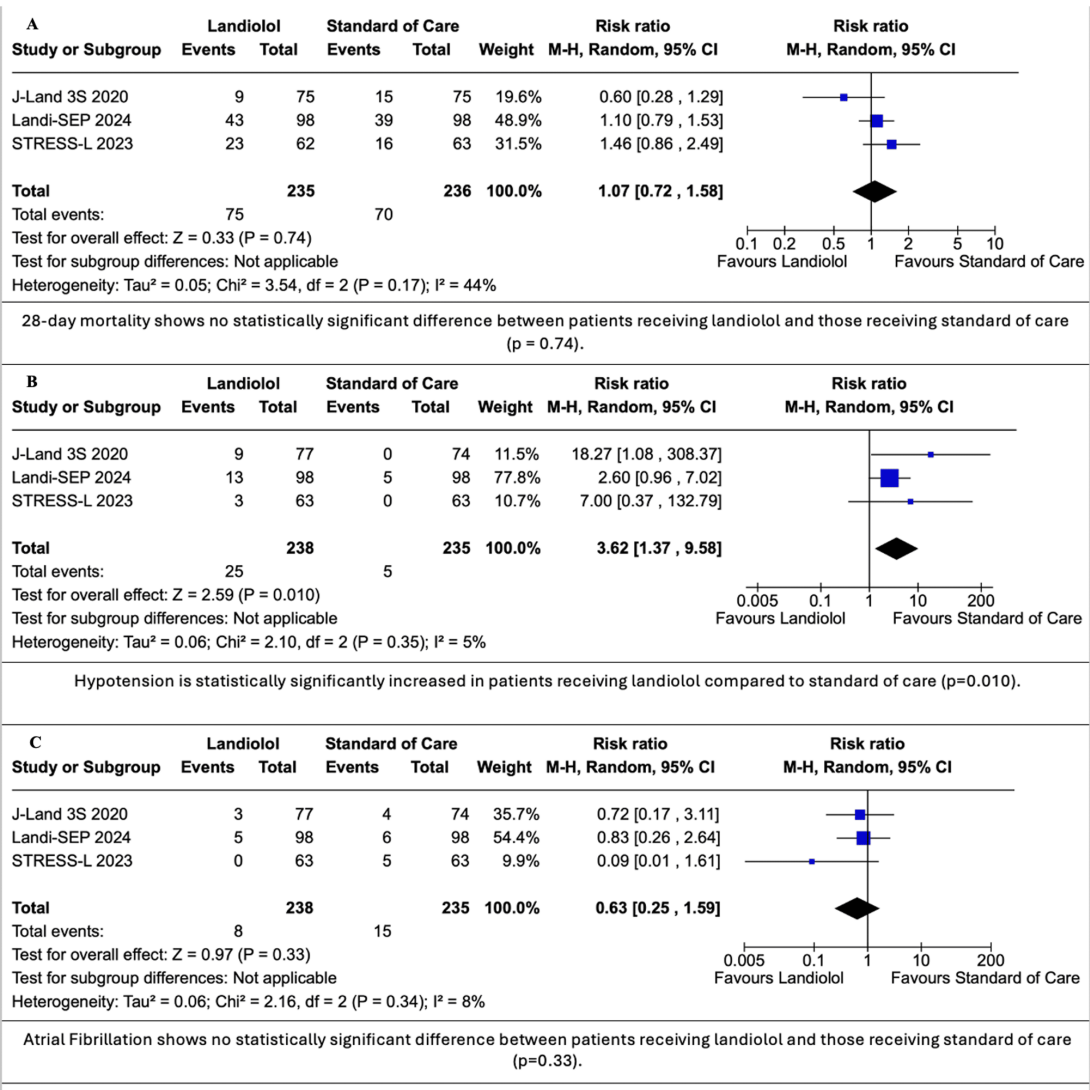
Forest plots for 28-day mortality, hypotension and AF Data were obtained from studies [3-5]. CI: confidence interval, AF: atrial fibrillation

**Figure 4:**
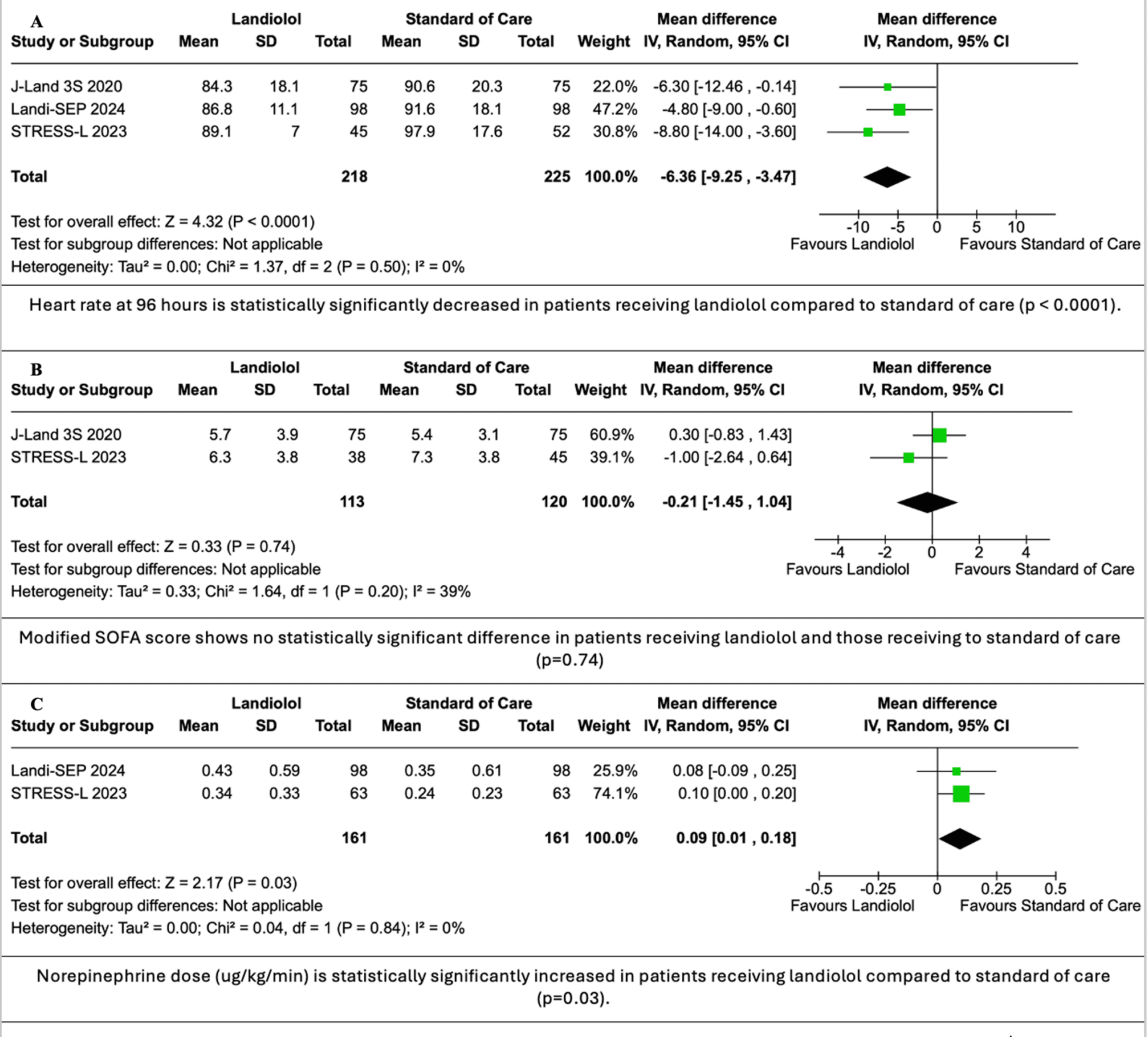
Forest plots for HR at 96 hours, modified SOFA score and norepinephrine dose Data were obtained from studies [3-5]. CI: confidence interval, SOFA: Sequential Organ Failure Assessment, HR: heart rate

The use of landiolol in sepsis and SS patients showed no statistically significant difference in 28-day mortality with landiolol compared to STC (RR: 1.07; 95% CI: 0.72, 0.72,1.58; p = 0.74; I^2^ = 44%, A in Figure 3), while HR at 96 hours, the therapy goal, was significantly decreased (MD: -6.36 bpm; 95% CI: -9.25, -3.47; p < 0.0001; I^2^ = 0%, A in Figure 4). All RCTs contributed to the primary outcomes.

Secondary Outcomes

Hypotension was significantly increased in the landiolol group compared to STC (RR: 3.62; 95% CI: 1.37, 9.58; p = 0.010; I^2^ = 5%, B in Figure 3). AF had no significant reduction with landiolol when compared to STC (RR: 0.63; 95% CI: 0.25, 1.59; p = 0.33, I^2^ = 8%, C in Figure 3), as was the modified SOFA score (MD: -0.21; 95% CI: -1.45, 1.04; p = 0.74; I^2^ = 39%, B in Figure 4). Norepinephrine doses were statistically significantly increased when comparing the landiolol group to STC (MD: 0.09; 95% CI: 0.01, 0.18; p = 0.03; I^2^ = 0%, C in Figure 4). All RCTs contributed to all secondary outcomes except for modified SOFA score [4,5] and norepinephrine doses [3,4].

Given the small number of included studies and participants, the limited data contributing to each outcome, and the wide CIs in some endpoints, these findings should be interpreted cautiously.

Discussion

This MA evaluated the efficacy and safety of landiolol, an ultra-short-acting β1-selective blocker, in patients with sepsis and SS. The findings provide important insights into the potential role of landiolol in this critically ill population, although the results highlight a complex balance between benefits and risks.

Primary Outcomes

Landiolol effectively reduced HR in sepsis and SS patients with persistent tachycardia, achieving a statistically significant mean decrease compared to standard therapy (p < 0.0001). This reduction aligns with its pharmacological target and was consistently observed across studies. However, the magnitude of HR reduction, approximately 6 bpm, may not be sufficient to produce meaningful clinical effects. Prior research suggests that reductions exceeding 10 bpm are more likely to improve survival in sepsis. In this analysis, organ function, as assessed by the SOFA score, showed no significant improvement (p = 0.74), highlighting a possible disconnect between modest HR control and clinical benefit.

The primary endpoint of 28-day mortality, however, did not demonstrate a statistically significant reduction in the landiolol group compared to the STC group (p = 0.74). This aligns with findings from three LANDI-SEP, STRESS-L, and J-Land 3S trials [3-5], where no mortality benefit was observed. The evidence for landiolol, however, remains inconclusive. Notably, heterogeneity (I² = 44%) indicates variability across studies, which may be due to differences in study design, patient populations, and treatment protocols.

Secondary Outcomes

Contrary to expectations, landiolol did not significantly reduce the incidence of AF (p = 0.33). This finding is consistent with the J-Land 3S, LANDI-SEP, and STRESS-L trials [3-5], which reported comparable AF rates between groups. While β-blockade has been associated with a reduction in AF in other settings, the lack of effect here may reflect differences in patient characteristics or the underlying pathophysiology of sepsis-related AF.

Hypotension was significantly increased in the landiolol group compared to STC (p = 0.010). This suggests an elevated risk that warrants careful clinical interpretation. However, the included trials did not consistently report the severity of hypotension, such as the need for vasopressors, escalation of therapy, or clinical outcomes directly attributed to hypotensive events. Future studies should provide more granular data to assess the clinical impact of this adverse effect.

Norepinephrine doses were statistically significantly higher in the landiolol group than in the STC group (p = 0.03). Although statistically significant, this increase may reflect minor adjustments typical in the dynamic management of SS, where titration of vasopressors is frequently required. The MD of 0.09 µg/kg/min lies within typical clinical adjustment ranges and may not be sufficient to affect outcomes unless correlated with sustained high-dose requirements or organ dysfunction [11].

Interpretation of Findings

The potential harm observed in the STRESS-L trial is noteworthy and warrants further examination in the context of our pooled analysis. While the STRESS-L trial suggested increased mortality and adverse effects, our pooled data on mortality and hypotension did not demonstrate statistically significant harm. This discrepancy highlights the importance of contextual interpretation, particularly in relation to trial design, patient selection, and outcome definitions. Additionally, although we compared landiolol and esmolol in our discussion, the lack of direct head-to-head trials limits the ability to draw definitive conclusions about the drug-specific effects. Variations may influence differences in trial outcomes in population characteristics, clinical settings, or β-blocker pharmacodynamics.

Moreover, while landiolol effectively reduced HR, this did not translate into improvements in SOFA score or AF incidence. Possible explanations for this dissociation include limited sample sizes, the short duration of follow-up, or heterogeneity in underlying disease severity. Mortality outcomes were evaluated globally; however, it is plausible that specific subgroups, such as patients with a baseline HR greater than 110 bpm, may benefit more from β-blocker therapy. Future analyses should further explore these subgroups. Additionally, the limited number of included studies reduces the power to detect rare events and precludes formal assessments of publication bias.

Beyond HR control, landiolol may exert additional physiological benefits that warrant consideration in the context of SS. Experimental and early clinical data suggest that β1-selective blockade with landiolol may attenuate the harmful effects of sympathetic overdrive, including excessive inflammatory cytokine release, microcirculatory disturbances, and myocardial oxygen supply and demand mismatch. These mechanisms could potentially improve sepsis-related organ dysfunction independent of HR reduction. However, the current pooled data did not demonstrate consistent improvements in surrogate outcomes such as the SOFA score or the incidence of new-onset AF. This dissociation between HR control and broader clinical endpoints may reflect limitations in study design, variability in patient severity, or the complex, multifactorial nature of septic cardiomyopathy. Therefore, while mechanistically promising, these pleiotropic effects remain speculative and should be investigated in larger, biomarker-driven randomized trials.

Safety Concerns

A critical finding of this study was the significant increase in hypotension in the landiolol group compared to the STC group (p = 0.010). Hypotension-related adverse events were frequently reported in the J-Land 3S and LANDI-SEP trials [3,5], and this consistent trend raises concerns about the safety profile of landiolol, particularly in a population already vulnerable to hemodynamic instability. This finding highlights the importance of careful patient selection and close hemodynamic monitoring during landiolol therapy.

The premature interruption of a trial not only occurred with esmolol but also with landiolol in the STRESS-L trial [4], which was terminated in December 2021 at the advice of the independent data monitoring committee. This decision was based on the observation that landiolol was unlikely to demonstrate a benefit, and there was a signal of potential harm in the intervention group. The trial had planned to recruit 340 participants but stopped after enrolling 126 patients (37.1% of the target). The early termination was not based on formal futility calculations but instead on interim analysis data and recruitment feasibility concerns, which indicated potential harm associated with landiolol use (higher mortality observed in the landiolol group compared to STC). This may also account for the substantial heterogeneity observed in mortality outcomes and contributes to the high risk of bias associated with the STRESS-L trial [4].

Comparison With Other β-Blockers

Persistent tachycardia and high HRs have always been a challenge in the ICU context. The knowledge of these clinical findings reflects concerns about the patient's hemodynamics. It leads to much research interest, as short-acting β1-selective adrenergic blockers tend to have fewer hypotensive effects and become a promising therapeutic, especially in sepsis and SS patients, a population with low blood pressure levels associated with inflammatory triggers. Previous trials with esmolol, another short-acting β1-selective adrenergic blocker, presented some interesting findings that were also uncertain. Landiolol is described in the literature as being more selective, with a rapid onset of action, metabolism independent of liver and kidney function, minimal negative inotropism, and higher potency at lower doses compared to esmolol [14]. A table outlining the differences between landiolol and esmolol is provided in the Appendices.

Several studies have investigated the use of esmolol, a short-acting β1-selective adrenergic blocker, for managing tachycardia in patients with sepsis and SS. The beginning of research in this field was demonstrated by Morelli et al. (2013) [8]. The study found that esmolol effectively reduced HR to target levels without increasing the need for vasopressors. Additionally, there was a significant reduction in 28-day mortality among patients receiving esmolol compared to the control group. Liu et al. 2019 [15] reported that esmolol administration was associated with decreased HR and 28-day mortality. However, the benefits varied depending on the initial HR, suggesting that patients with HRs between 110 and 120 bpm might benefit more from esmolol treatment. Levy et al. (2021) [16] were interrupted due to safety concerns, such as an increased risk of hypotension and a decreased cardiac index.

Clinical Implications

This MA is the first to specifically examine landiolol as a standalone intervention in patients with sepsis and SS, distinguishing it from prior reviews that combined various β-blockers. Previous MAs suggested potential survival benefits for ultra-short selective β1-blockers, esmolol, in sepsis [17-19]. However, recent MAs have reported inconsistent effects of ultrashort-acting β-blockers in septic patients. A 2021 MA by Hasegawa et al. [20] showed that both esmolol and landiolol significantly reduced HR and 28-day mortality in patients with sepsis and SS. On the other hand, a 2024 MA by Vásquez-Tirado et al. [21] found no mortality benefit, though HR reduction remained significant. Similarly, a 2024 MA by Alexandru et al. [22] reported no significant association between β-blocker use and short-term mortality. These mixed findings highlight uncertainty surrounding mortality outcomes, despite consistent HR control in some studies.

This targeted approach helps isolate the efficacy and safety profile of landiolol, offering more precise guidance for clinical decision-making. The results of this MA suggest that while landiolol effectively reduces HR, it does not confer significant improvements in mortality or organ function and is associated with an increased risk of hypotension. These findings underscore the importance of individualized patient selection, particularly for those who may benefit from HR control without experiencing adverse hemodynamic effects. This is an expert opinion regarding the importance of hemodynamic risk stratification, using cardiac output, fluid responsiveness, and vasopressor requirements in guiding therapy for sepsis or SS [23].

Future Directions

Future research should focus on identifying subgroups of SS patients who might derive the most significant benefit from landiolol, such as those with hyperdynamic circulation or persistent tachycardia despite optimized resuscitation. Additionally, combining landiolol with adjunctive therapies, such as inotropes or advanced hemodynamic monitoring, should be explored to mitigate its adverse effects.

Limitations

This MA has several limitations that may affect the robustness of its conclusions. Variability in study designs, patient populations, and methodologies across J-Land 3S, STRESS-L, and LANDI-SEP [3-5] introduces heterogeneity, which may influence effect estimates. The open-label nature of the trials also raises the risk of bias in outcome assessment. Small sample sizes, particularly in subgroup analyses, limit the statistical power to detect rare events. Moreover, with fewer than 10 studies included, assessing publication bias reliably was not feasible.

Notably, the STRESS-L trial [4] negatively impacted 28-day mortality outcomes and was terminated early due to safety concerns; a leave-one-out sensitivity analysis confirmed improved mortality results when STRESS-L [4] was excluded. It should be emphasized that this sensitivity analysis reflects a statistical change in pooled estimates and does not establish causality or justify excluding the trial from the analysis. Moreover, STRESS-L's [4] early termination without a formal futility threshold may exaggerate or obscure true treatment effects. The risk of bias assessment identified STRESS-L [4] as having a high risk of bias, particularly in adherence to interventions and reporting of outcomes.

## Conclusions

While landiolol was generally tolerated, its use is conditional and depends on the patient’s hemodynamic status. The significant increase in hypotension and norepinephrine requirements raises important safety concerns, particularly highlighted by findings from the STRESS-L trial. Therefore, broad recommendations for landiolol use in SS are premature. Further safety evaluation is necessary before widespread adoption can occur. Given the limited RCT data available, these conclusions should be interpreted with caution. Future studies using stratified or biomarker-guided approaches are needed to identify better patients who might benefit most from landiolol therapy.

## Appendices

Search strategy

MEDLINE and Embase

(landiolol OR ‘beta blocker’ OR "Adrenergic beta-Antagonists"[Mesh])

AND

('septic shock' OR sepsis OR "Shock, Septic"[Mesh])

AND

(tachycardia OR tachyarrhythmia OR ‘high heart rate’ OR AF OR ‘atrial fibrillation’ OR "Atrial Fibrillation"[Mesh])

AND 

("randomized controlled trial"[pt] OR "controlled clinical trial"[pt] OR randomized [tiab] OR placebo[tiab] OR "drug therapy"[sh] OR randomly[tiab] OR trial[tiab] OR groups[tiab])

Equivalent terms were adjusted for usage in Embase.

Cochrane

(landiolol OR ‘beta blocker’)

AND

('septic shock' OR sepsis)

AND

(tachycardia OR tachyarrhythmia OR ‘high heart rate’ OR AF OR ‘atrial fibrillation’)

Table 3

**Table 3:** Comparison of landiolol and esmolol [14]

Feature	Landiolol [14]	Esmolol [14]
β1-selectivity	Highly selective	Moderately selective
Onset of action	Very rapid (1–2 min)	Rapid (2–5 min)
Half-life	~4 minutes	~9 minutes
Metabolism	Metabolized by plasma esterases, independent of liver/kidney function	Metabolized by plasma esterases
Potency	Higher potency, allowing lower doses for similar effect	Lower potency
Cardio depressive effects	Minimal negative inotropism	Moderate negative inotropism
Hemodynamic stability	Less hypotension and bradycardia	Greater risk of hypotension and bradycardia
Clinical use	Atrial fibrillation, atrial flutter, and supraventricular tachycardia; preferred in critical care due to safety profile	Intraoperative tachycardia, supraventricular tachycardia, and hypertensive crises
Dosing	Lower doses required due to high potency (1–40 µg/kg/min)	Higher doses needed (50–300 µg/kg/min)
Safety in HF patients	Preferred due to minimal cardiac suppression	Higher risk of worsening heart failure
